# Enrichment of putative plant growth promoting microorganisms in biodynamic compared with organic agriculture soils

**DOI:** 10.1093/ismeco/ycae021

**Published:** 2024-02-05

**Authors:** Felix Milke, Heberto Rodas-Gaitan, Georg Meissner, Vincent Masson, Meike Oltmanns, Morten Möller, Yvette Wohlfahrt, Boris Kulig, Alberto Acedo, Miriam Athmann, Jürgen Fritz

**Affiliations:** Department of Organic Farming and Cropping Systems, University of Kassel, D-37213 Witzenhausen, Germany; Department of Organic Farming and Cropping Systems, University of Kassel, D-37213 Witzenhausen, Germany; Department of General and Organic Viticulture, University of Geisenheim, D-65366 Geisenheim, Germany; BioDynamie Services, Les Crêts, 71250 Chateau, France; Forschungsring e.V., D-64295 Darmstadt, Germany; Department of Organic Farming and Cropping Systems, University of Kassel, D-37213 Witzenhausen, Germany; Department of General and Organic Viticulture, University of Geisenheim, D-65366 Geisenheim, Germany; Department of Agricultural and Biosystems Engineering, University of Kassel, D-37213 Witzenhausen, Germany; Biome Makers Inc., Davis, CA 95618, United States; Department of Organic Farming and Cropping Systems, University of Kassel, D-37213 Witzenhausen, Germany; Department of Organic Farming and Cropping Systems, University of Kassel, D-37213 Witzenhausen, Germany

**Keywords:** microbiome, agriculture, biodynamic, organic, biodynamic preparation, soil health, biofertilizer, soil, biological amendment

## Abstract

The potential of soils to maintain biological productivity, defined as soil health, is strongly influenced by human activity, such as agriculture. Therefore, soil management has always been a concern for sustainable agriculture and new methods that account for both soil health and crop yield must be found. Biofertilization using microbial inoculants emerges as a promising alternative to conventional interventions such as excessive mineral fertilization and herbicide use. Biodynamic preparations used as a central part of biodynamic agriculture have various effects on soil properties, such as microbial biomass and respiration. We conducted several biomarker experiments to infer the effect of biodynamic preparations on soil prokaryotic and fungal communities and compared results to organic management. Potential plant growth promoting amplicon sequence variants were quantified using a commercial database based on their taxonomic identity. We found significantly higher numbers of putative plant growth promoting amplicon sequence variants in biodynamically compared with organically treated soils. Furthermore, prokaryotic amplicon sequence variants enriched in biodynamic preparations were found in higher numbers in biodynamically treated soils, indicating successful colonization after treatment. Experiments were conducted at three locations in Germany and 21 locations in France covering different crops and soil types. Altogether, our results indicate that biodynamic preparations can act as biofertilizers that promote soil health by increasing the abundance of plant growth promoting microorganisms.

## Introduction

Large-scale ecosystem degradation is a consequence of agricultural intensification because of the application of pesticides, consumption of water storages, and soil degradation, which is a rising issue with an increasing global population [1, 2]. To counter this development, low-input systems such as organic or biodynamic farming emerged as sustainable alternatives to conventional farming strategies [3]. Both farming strategies share similar principles, such as refraining from the use of synthetic fertilizers or pesticides. However, biodynamic agriculture favors the use of composts, the integration of livestock, and the reduction of external inputs to a greater extent than organic agriculture. One essential difference between organic and biodynamic crop farming is the application of so-called biodynamic preparations that were proposed in the beginning of the 20th century by Rudolf Steiner [4], the founder of biodynamic agriculture. These preparations are either applied in the field on soil or crops (“field preparations”) or on stable manure (“compost preparations”). The compost preparations consist of different wild plants fermented in combination with different organs of ruminants. The field preparations consist of fermented manure or silica flour (preparation BD500: horn manure and preparation BD501: horn silica) stored in cow horns and burrowed for 6 months in soils. After fermentation, the highly diluted products are sprayed on the fields where they showed an improvement of multiple parameters: soil aggregate stability [5], higher soil activity and nutrient availability [6, 7], higher vegetable or cereal grain yield [6-9], higher content of secondary plant compounds [10, 11], and promotion of the germination of seeds in the following generation [12]. Despite numerous crop beneficial effects that could be associated with the use of biodynamic preparations, some cases report no significant differences between agricultural managements with and without biodynamic preparations [13, 14]. Long-term observations from several experimental sites by Raupp and König [15] indicate that biodynamic preparations have a system regulating effect: they found that under unfavorable growth conditions crop yield was increased, whereas under good growth conditions with high to very high nutrient supplies crop yield was not affected or even reduced when treated with biodynamic preparations.

Biodynamic preparations have as low application rates as 100 g ha^−1^ of fermented manure for horn manure and 4 g ha^−1^ of quartz powder for horn silica, hence their effect cannot simply be attributed to nutrient supply. Horn manure is applied to moist soil in autumn and spring in large drops. Horn silica is sprayed onto the leaves in a fine mist during the growing season. Both are applied one to four times a year. There are different explanatory models to describe the effect of the preparations on crop management. For example, in the production of horn manure preparations, the microbially mediated slow fermentation under oxygen-deficient conditions in the soil can produce signaling molecules such as carbohydrates and peptides to which microbes respond even at very low concentrations [16]. This could lead to increased microbial activity in the rhizosphere [17-19] or stimulate natural plant defenses [20, 21]. Another complementary explanation for the potential mode of action of the preparations could be microbially mediated plant growth promoting effects. For example, bacterial strains that produce indole acetic acid (IAA) were detected in horn manure preparations [22]. According to Spaccini *et al.* [16], horn manure also contains lignin residues with IAA-like activity. Besides that, auxin-like and gibberellic acid-like effects were found in horn manure and horn silica preparations, respectively [17].

It is hypothesized that plant beneficial effects of biodynamic preparations can be induced by an enhancement of the symbiosis between plants and microbes either via the successful colonization of beneficial microbes present in the preparations [23], or by stimulating microbial activity in the soil with biolabile compounds [16]. Significant positive effects of horn manure and horn silica preparations on microbial respiration in soils [24] support the hypothesis of microbially mediated effects on plants. Furthermore, a recent analysis of soil microbiomes managed under different agricultural practices revealed a strong connection between management practice and microbial interaction structure, where especially biodynamic management increased microbial community stability by promoting more densely connected communities [25]. Hence, there is evidence that biodynamic preparations impact soil microbial communities that promote the observed effects on plant growth.

In the present study, we aimed to infer changes in the prokaryotic and fungal community compositions of agriculturally used soils associated with biodynamic field preparations (BD500 or BD500P (“P”: treated with additional preparation, see below) and BD501). We tracked the occurrence of amplicon sequence variants (ASV) enriched in biodynamic preparations in microbial communities of biodynamically managed soils to observe successful microbial colonization. Furthermore, we infer potential plant beneficial effects associated with the observed community changes. To do that, we assigned potential plant beneficial effects to taxonomic identities of microbial ASVs using a commercial database (Biome Makers) that we validated with an in-house database based on peer-reviewed publications. We aimed to analyze the following hypotheses:

(1) Biodynamic field preparations affect the microbial community composition of soils either via successful colonization of microorganisms enriched in field preparations or via biostimulation.
(2) The application of biodynamic field preparations increases the number of plant growth promoting microorganisms in soils.
(3) Biodynamic field preparations contain high proportions of plant growth promoting microorganisms.
(4) The increase of plant growth promoting microorganisms induced via biodynamic field preparations is transient.

We applied our approach to four different experimental setups to test our hypotheses, where we used a block design to analyze the effect of the biodynamic preparations on a broad spectrum of soils with various crops, at different locations in central Germany and France at two timepoints, and at selected locations also in a 15-week time series to follow the dynamics of soil colonization and potential plant beneficial effects.

## Materials and Methods

### Experimental sites and setups

In total, we took 254 soil samples from three agricultural or viticultural experimental sites in Germany (Frankenhausen, Geisenheim, Darmstadt) and 21 practical agricultural or viticultural farms in France throughout the vegetation period in 2021. We covered a broad range of different farming setups, including various crops, soil types, and climatic conditions in central Germany and in France. The rational of this experimental design was to analyze the effect of the biodynamic field preparations on soil microbial communities under realistic settings in a range of typical agroecosystems in central Europe.

Since biodynamic crop farming differs from organic crop farming mainly in the application of biodynamic preparations, we used organic crop management (BD−) as control at Frankenhausen, Geisenheim, and France. At Darmstadt, we analyzed the effect of increased application intensity and used extensive biodynamic management (BD+) as control. That is, we compared the typical practice of three spray treatments of horn manure and horn silica (BD++) each with an extensive setting where we used only one treatment per preparation (Supplementary Table S1).

**Figure 1:**
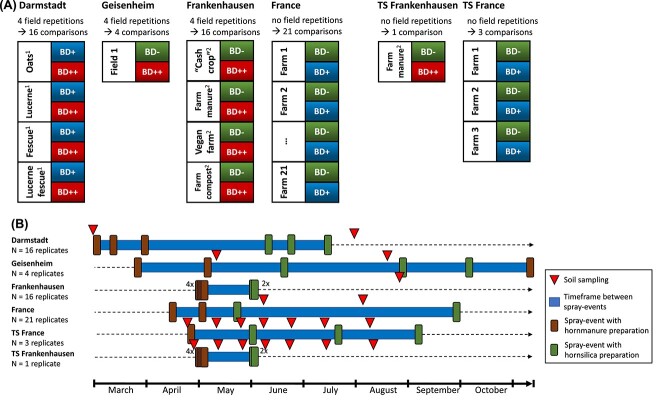
(A) Block design of experimental fields. Each field in Darmstadt, Geisenheim, and Frankenhausen was split into four blocks. In Geisenheim and Frankenhausen, a block was treated half with biodynamic preparations (BD++) and half without as control (BD−). In Darmstadt, we analyzed the effect of application intensity using single application (BD+) compared with three applications of biodynamic preparations (BD++). In France and time series experiments (TS), fields contained only one block that was split in half without and with BD (BD− vs. BD+). (B) Timeline of experiments, including timepoints of biodynamic preparation treatments (spray events are marked with rectangles according to legend) and timepoints of soil sampling (marked with triangles). Timeframe between first and last spray event is highlighted with a blue line. German cities are displayed by names, whereas France cities were grouped into the France experiment (see Supplementary Table S2 for detailed locations). A number of replicates are listed to the left.

At all sites, application of the biodynamic field preparations vs. a control was tested, integrated into various experimental setups which are described in Fig. 1A and in the supplementary material. At Frankenhausen and Darmstadt, application of biodynamic field preparations was integrated into running field experiments. We implemented a two factorial split-plot design with four different organic farming systems (Frankenhausen) or four different precrops (Darmstadt) as main plot. The application of the biodynamic preparations was compared within subplots. Fields from practical farms or vineyards in France were split in half with one half being treated with biodynamic preparations and the other as control (Fig. 1A). Setup and management of all sampled sites are described in Table 1 and with more detail in the supplementary information.

**Table 1:** Detailed description of experimental sites and applied biodynamic preparations. Additional information on experimental sites in France can be found in Supplementary Table S2.

**Location**	**Crop**	**Soil type**	**Sampling dates/weeks after first spray**	**Biodynamic preparation type and origin**	**Sample-number**	**BD since**
**Darmstadt** **(Germany)**	Oat, rye, tall fescue	Sandy	T0: 1 March 2021 0 weeks T1: 3 August 2021 19 weeks	BD500 Darmstadt BD501 Darmstadt	16 × 2 timepoints	2019
**Geisenheim** **(Germany**	Vine	Sandy loam	T0: 10 May 2021 7 weeks T1: 18 August 2021 21 weeks	BD500 Geisenheim BD501 Geisenheim	4 × 2 timepoints	2006
**Frankenhausen** **(Germany)**	Wheat, spelt, oat	Loess	T1: 25 August 2021 16 weeks	BD500P Cluny BD501 Cluny	16 × 1 timepoint	2021
**France**	13× vine, rye, 2× chickpeas, barley, garlic, wheat, flax, sunflower	13× clay, 4× loam, 4× sandy loam	T0: 7 June 2021 ~7 weeks T1: 4 August 2021 ~15 weeks	BD500P Cluny BD501 Cluny	21 × 2 timepoints	2001–2021
**TS France**	Vine	Clay	24 April 2021 (0 weeks) 10 May 2021 (2 weeks) 24 May 2021 (4 weeks) 7 June 2021 (6 weeks) 23 June 2021 (8 weeks) 12 July 2021 (11 weeks) 8 August 2021 (15 weeks)	BD500P Cluny BD501 Cluny	3 × 7 timepoints	2021
**TS Frankenhausen** **(Germany)**	Cereals	Loess	27 April 2021 (0 weeks) 11 May 2021 (2 weeks) 25 May 2021 (4 weeks) 8 June 2021 (6 weeks) 22 June 2021 (8 weeks) 13 July 2021 (11 weeks) 10 August 2021 (15 weeks)	BD500P Cluny BD501 Cluny	1 × 7 timepoints	2021

Treatments with biodynamic preparations varied between farms in terms of preparations used and timepoints of spraying (Fig. 1B and Table 1). Soil communities were sampled twice per location (except Frankenhausen and both time series) at different timepoints during the growth period, from 1 day before the spray treatment up to 21 weeks after the first spray treatment. First soil samples were taken between March and June (T0), whereas second sampling was done in August (T1). Biodynamic soil samples were taken at Frankenhausen only in August because of logistic reasons. We further conducted a time series at three locations in France and one in Germany, where we sampled right before the first spray treatment and 2, 4, 6, 8, 11, and 15 weeks thereafter. A detailed description of each experimental setup is provided in Table 1 and in the supplementary material. For each soil sample, we mixed eight punctures of soil down to a depth of 13 cm.

We also sampled biodynamic preparations from various farms in Germany (Darmstadt, Bad Vilbel, Velden, Zülpich) and commercial preparations from BioDynamie Services (Chateau, France). The latter were applied at Frankenhausen and at all locations in France, the preparations from Bad Vilbel were applied at Geisenheim and are therefore denoted as “Geisenheim” throughout this article, and at Darmstadt the own preparations were used. The preparations from Velden and Zülpich were not applied at the experimental sites but we included them in our analysis to increase the variety of preparations and make our conclusions more generalizable.

### Biodynamic preparations

Biodynamic preparations are typically produced and applied locally. However, as their formulation follows complex recipes they are often produced and distributed by specialized manufacturers. To cover both scenarios, the experimental sites received their biodynamic preparations either from a manufacturer (BioDynamie Services, Chateau, France) or were produced locally: all experimental soils in France and the soils at Frankenhausen (Germany) were treated with preparations from BioDynamie Services, whereas soils at Geisenheim and Darmstadt were treated with locally produced preparations.

Horn manure (BD500): cow dung is put into a cow horn, buried in the soil in autumn and extracted after 6 months in spring. 100 g ha^−1^ of the fermented dung is stirred in 37°C water for 1 h. The amount of water used depends on the liquid used per ha by the spraying technique and ranges from 50 to 100 l ha^−1^. Horn manure is applied in large drops, especially in spring at the start of the growing season and applied directly onto the moist soil, if possible.

Horn manure prepared (BD500P): production is the same as for horn manure (BD500), except it is further treated with the biodynamic compost preparations. After the horn manure has been taken out of the horn in spring, it is placed in ~50 l containers. These containers with horn manure are treated like compost with biodynamic compost preparations that contain fermented medicinal herbs (e.g. yarrow, chamomile).

Horn silica (BD501): crystalline quartz is pulverized to a fine powder. The quartz flour is filled into cow horns with ~30 ml water. Once the quartz flour has settled the water is removed. The cow horn is subsequently buried in the soil in spring and dug out in autumn after 6 months. Of the quartz flour, 4 g ha^−1^ is stirred in 37°C warm water for 1 h. The amount of water used depends on the spraying technique and its liquid requirement per ha. Horn silica is sprayed onto the leaves in a fine mist. The time of application can therefore start at the time when the leaves are fully developed, and application can be continued throughout the entire vegetation period. 

### DNA extraction and library preparation

All samples were sent to the Biome Makers laboratory in Valladolid, Spain, for DNA extraction. The DNeasy PowerLyzer PowerSoil kit from Qiagen was used for nucleotide extraction using the BeCrop® platform (patent publication number: WO2017096385, Biome Makers). The V4 region of the 16S rRNA gene and the ITS1 region (BeCrop custom primers: patent WO2017096385) were analyzed to retrieve prokaryotic and fungal microbial communities from bulk soils, including roots and associated rhizosphere. The libraries for ITS and 16S rRNA were prepared using a two-step PCR protocol as described by Liao *et al.* [26] and Gobbi *et al*. [27]. All samples were sequenced on an Illumina MiSeq instrument (Illumina, San Diego, CA, USA) using 2 × 251 paired-end reads.

### Bioinformatics

After sequencing, reads were processed by first removing primers from paired end reads using Cutadapt [28] and trimmed reads were merged with a minimum overlap of 100 nucleotides. Next, sequences were quality filtered with an Expected Error threshold of 1.0 [29]. Quality filtered reads were iteratively clustered into ASVs using Swarm [30]. De novo chimeras and remaining singletons were removed by applying the USearch pipeline [31], and taxonomy was assigned for each ASV using a global alignment with 97% identity against SILVA138.1 for 16S rRNA sequences and UNITE8.3 for ITS sequences [32, 33].

### Potential plant growth promoting effects

Abundance data on plant growth promoting prokaryotes and fungi were inferred by Biome Makers Inc. (California, USA) who patented a method called BeCrop^Ⓡ^ indices to infer agronomically relevant functional information from taxonomies, comparable to Tax4Fun2 [34] and FAPROTAX [35]. BeCrop indices are patented indicators to assess health status of soils based on metagenomic data as described by Acedo *et al.* [36]. Briefly, these indicators assess relevant traits related to soil health ranging from metabolic potential to biocontrol and hormones estimations. Detailed descriptions of a subset of BeCrop indices relevant to this study are provided in Supplementary Table S4. The underlying databases infer stress adaptation based on several mechanisms: abscisic acid (ABA), 1-aminocyclopropane-1-carboxylate (ACC) deaminase, exopolysaccharide (EPS) production, heavy metal solubilization, salicylic acid, salt tolerance, and siderophore production. Additionally, they deliver potential hormone production based on cytokinin, gibberellin, and IAA production. All potential mechanism abundances are based on the combination of relevant prokaryotic and fungal abundances and scaled to an index from 1 to 6 with 1 indicating low abundance and 6 indicating high abundance in the respective soil sample. Biome Makers supplied us also with unscaled relative abundances of microbes that have potential plant growth promoting effects in the biodynamic preparations.

To verify their databases, we created an additional database based on a literature review about plant growth promoting effects induced by prokaryotes and fungi (Supplementary Data—Excel sheet “Literature Review Prokaryotes/Fungi”). We inferred relative abundances of all potentially plant growth promoting organisms based on taxonomic level of genus, as the phylogenetic resolution of amplicon studies often struggles with delineation on species or even subspecies level [37]. We created a linear model based on ITS and 16S abundances to predict index-values using least squares regression. The models were inferred for hormone production and stress adaptation and yielded good fits (hormone production: Adj. *R*^2^ = 0.353; stress adaptation: Adj. *R*^2^ = 0.346) (Supplementary Fig. S1). These results showed how the workflow of Biome Makers index inference works, but also that their databases are superior to the limited literature review that we conducted for their verification. Therefore, we continued our analyses with the Biome Makers indices as described below.

### Assessing colonization from microbes enriched in biodynamic preparations

We defined ASVs to be associated with biodynamic preparations if they had relative abundances above 0.5% in the biodynamic preparation samples, because we assume that the preparations contain relevant numbers of soil associated ASVs as they are fermented within the soil. We tested several abundance thresholds to define enriched organisms (0.1%, 0.5%, 1%) and picked an intermediate value of 0.5% as there was no large difference in the outcome of colonization success in the tested range of thresholds. We assume that higher values will strongly decrease detection sensitivity, whereas lower values might increase the proportion of soil-associated organisms in this analysis. A colonization success was apparent when soils treated with biodynamic preparations had higher abundances of ASVs associated to biodynamic preparations compared with the untreated soil samples of the same block.

### Statistical analysis

All statistical analyses were conducted in R (version 4.2.2). For the statistical analysis of the Biome Makers index-values we tested the data set for normal distribution with the Kolmogorov–Smirnov test and for homogeneity of variances with the Levene test. Data points falling above three times the interquartile range, above or below the highest or lowest quartile of the outlier box plot, were removed as outliers. We used paired *t*-test to infer significant differences between treatments for normally distributed data and paired Wilcoxon test for not normally distributed data. Treatment and control for each block were analyzed as paired measurements. All test statistics are mentioned in the text or in the supplementary data. For NMDS count tables were transformed to relative abundances and Hellinger transformed using the “decostand” function before computing Bray–Curtis dissimilarities between samples using the “vegdist” function from the vegan package (version 2.6-4). We used the pheatmap package (version 1.0.12) to create a heatmap of relative abundances of putative PGP microbes in biodynamic preparations.

## Results

### Distinct soil microbiomes across experimental setups

We sequenced prokaryotic (16S rRNA gene) and fungal (ITS) communities of 254 soil samples and 20 biodynamic preparations (of which six ITS samples did not yield sufficient read counts), resulting in a total of 532 samples (254 × 16S rRNA + 254 × ITS soil samples and 14 × 16S rRNA + 10 × ITS biodynamic preparation samples). 16S rRNA gene samples were sequenced to an average of 32 616 counts (s.d. 23 875 counts) and ITS samples to an average of 53 899 counts (s.d. 40 995 counts) after bioinformatic processing. Fungal communities had much lower average number of ASVs per sample (63 ASVs/sample of total 2025 ASVs in the data set) than prokaryotic communities (1434 ASVs/sample of total 55 679 ASVs in the data set).

The taxonomic composition of prokaryotic communities on class level was highly similar between locations, timepoints, and farming practices (Supplementary Fig. S2A). Most ASVs belonged to Actinobacteria, Alphaproteobacteria, and Nitrososphaeria, comprising together more than 50% of community composition. Prokaryotic samples differed more distinctly on higher taxonomic levels, and their ASV compositions clustered strongly according to locations (Supplementary Fig. S3A). Farming practice and sampling time had only minor effects on community differences.

Fungal communities, however, expressed higher variability between locations and sampling time (Supplementary Fig. S2B). Variability between farming practices was low even on ASV level compared with the community differences associated with location and sampling time (Supplementary Fig. S3B). Even though samples from France were taken from different farms in different regions (Table 1 and Supplementary Table S2), their prokaryotic and fungal communities were very similar and did not express the same variability as samples located in Germany.

### Colonization of microorganisms through biodynamic preparations

**Figure 2:**
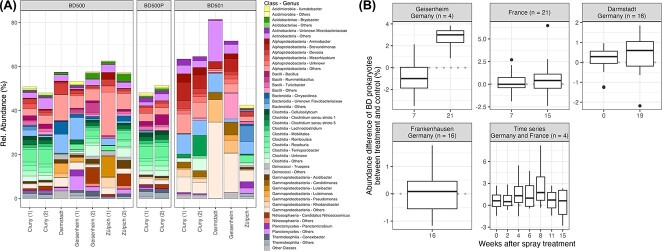
Prokaryotic communities enriched in biodynamic preparations and their abundance in soils. (A) Composition of prokaryotes enriched in biodynamic preparations from various locations and preparation types. ASVs are defined to be enriched in biodynamic preparations if they have relative abundance higher than 0.5%. Taxonomic assignment is displayed at genus level and color coded according to the legend. (B) Abundance difference of prokaryotic ASVs enriched in biodynamic preparation between treatment and control soils. Positive values indicate higher abundance of ASVs in treated soils.

The prokaryotic communities differed strongly between preparations with and without manure. While communities associated with preparations of manure were highly enriched in organisms from the taxonomic class Clostridia, horn silica preparations were enriched in various genera of Gammaproteobacteria. The different locations also showed clear differences in prokaryotic community composition that even varied within the same preparation type and the same location (e.g. horn manure preparation from Zülpich, Germany) (Fig. 2A and Supplementary Fig. S4). Similar to the soil communities, prokaryotic communities in the biodynamic preparations also contained high relative abundances of Alphaproteobacteria but were enriched in different genera compared with the soil samples. This was true for genera from all classes: the ASVs that we defined to be enriched in biodynamic preparations were only marginally abundant in the soil samples themselves.

However, we found significantly higher abundance of prokaryotic ASVs that were enriched in the preparations in biodynamically treated soils as compared with the control (nonparametric paired test: 16S rRNA *P*-value < 10^−3^, *V* = 5401) but not of fungal ASVs (ITS *P*-value = 0.083, *V* = 4640). To assess their colonization patterns in the soil communities after the spray treatment, we calculated the difference between their abundance in the biodynamically treated and the untreated soils. Positive abundances indicate a successful colonization on treatment, whereas an abundance of zero or below indicates unsuccessful colonization. As soil samples were taken at different time intervals in each experimental trial, we analyzed the colonization success for each timepoint, displayed as weeks after the first spray treatment (Fig. 2B).

The results generally showed a positive trend with increasing time, especially in the Geisenheim and Darmstadt trials, with 0.5% and 3% higher relative abundances of prokaryotic ASVs enriched in biodynamic preparations in treated compared with untreated soils at T1. Samples from France, however, did not show substantial abundance differences between treatments and increased little with time. Soils in Frankenhausen were sampled 16 weeks after the first spray treatment; at this time, prokaryotes enriched in biodynamic preparations expressed no abundance differences between treatments.

The time series data showed a distinct pattern of colonization success with increasing differences between biodynamically and organically managed soils until 8 weeks after the first spray treatment and declining afterwards. Even though we found the strongest effect in the time series 8 weeks after the first spray treatment, the trials in Geisenheim and Darmstadt had increased the abundance of biodynamic preparation enriched prokaryotic ASVs 21, respectively, 19 weeks after treatment. Fungal communities varied much stronger between treatments and locations, expressing abundance differences between treated and untreated soils of up to 57% of fungal communities (Supplementary Fig. S5). As described before, fungal ASVs were not significantly enriched in treated soils as compared with untreated soils and we did not observe a clear pattern associated with weeks after the first spray treatment (Supplementary Fig. S5B).

The prokaryotic communities enriched in biodynamic preparations showed only a weak difference between samples from different countries, whereas the fungal communities expressed strong country-specific differences. The differentiation between preparations with and without manure was still prominent in fungal communities, but not as strong as in prokaryotic communities. Generally, prokaryotic and fungal communities both expressed higher variability between different preparations than within preparations (Supplementary Fig. S4).

Fungal communities that were enriched in biodynamic preparations expressed high abundances of ASVs that were present in soil samples, such as organisms from the genera *Mortierella* and *Pseudeurotium* (Supplementary Fig. S5A). They had a relatively low richness of only 17–45 ASVs per sample, whereas prokaryotic communities enriched in biodynamic preparations comprised 85–169 ASVs per sample and a high number of ASVs that were below the 0.5% abundance threshold.

### Potential plant growth promoting effects increased in biodynamically treated soils

**Figure 3:**
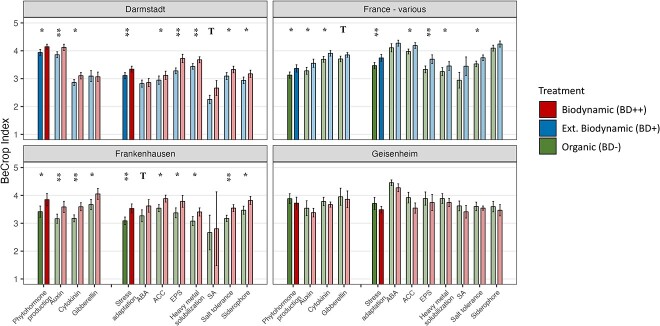
Quantitative analysis of putative plant growth promoting functions performed by soil microbial communities. Functional abundance is represented by the BeCrop index from Biome Makers and ranging from 1 to 6. Microbial functions that promote plant growth are separated by hormone production and stress adaptation. Individual functions are shown in light colors and functional groups are displayed in dark colors. Treatments are depicted by color according to the legend (see Table 1 for more details). Error bars represent standard errors and bar height shows average values. Symbols above bars represent statistical significance: *T* = *P*-value < 0.1, * = *P*-value < 0.05, ** = *P*-value < 0.01. Barplots are separated by experiment location into Darmstadt, France (various), Frankenhausen, and Geisenheim. See Supplementary Table S2 for more details about locations in France.

We evaluated 10 different PGPE that could be grouped in either microbial hormone production, such as cytokinin and auxin, or stress adaptation mechanisms, such as increased salt tolerance and heavy metal solubilization (Fig. 3). We describe these effects as potential PGPE to highlight that taxonomy-based analyses have limitations: taxonomy-based inference would fail when only certain strains of a taxon possess the functional genes for the assigned effects [38]. We define an increase in the individual effects as induced by the biodynamic preparations in soil if the biodynamic treatment expressed significantly higher PGPE values than the control treatment (Supplementary Table S3).

The horn manure and horn silica preparations (BD500P and BD501) that were used in the Frankenhausen trial led to significantly higher values of potential PGPEs for 10 out of 12 parameters (Fig. 3, Supplementary Table S3). The strongest effect was found in heavy metal solubilization, but also distinct differences in potential auxin and cytokinin production.

Treatments with the biodynamic spray preparations (BD500P and BD501) in the 21 experimental plots in France led to significantly higher values of potential PGPEs for 8 out of 12 parameters and for 10 effects the increase was greater than 5%. Here, the strongest effects were detected for ACC deaminase and EPS, both grouped into stress adaptation mechanisms that generally showed a highly significant effect.

The Darmstadt trial in which we investigated the spray frequency showed that three spray treatments of horn manure and horn silica resulted in 9 out of 12 significantly higher potential PGPEs compared with the control with one spray treatment and 9 effects were increased by more than 5%. The strongest difference of potential PGPEs between treatment and control was also found for EPS and hormone production (mostly auxin).

The Geisenheim trial stood out in this analysis as it yielded no significant differences, or even trends, in potential PGPEs between control and treatment. Even though no significant differences were found for potential PGPEs in Geisenheim, it is noteworthy that all 12 effects were lower in the preparation treatment.

All *P*-values and test statistics are reported in the Supplementary Data.

### Relative abundance of potential plant growth promoting organisms in preparations

**Figure 4:**
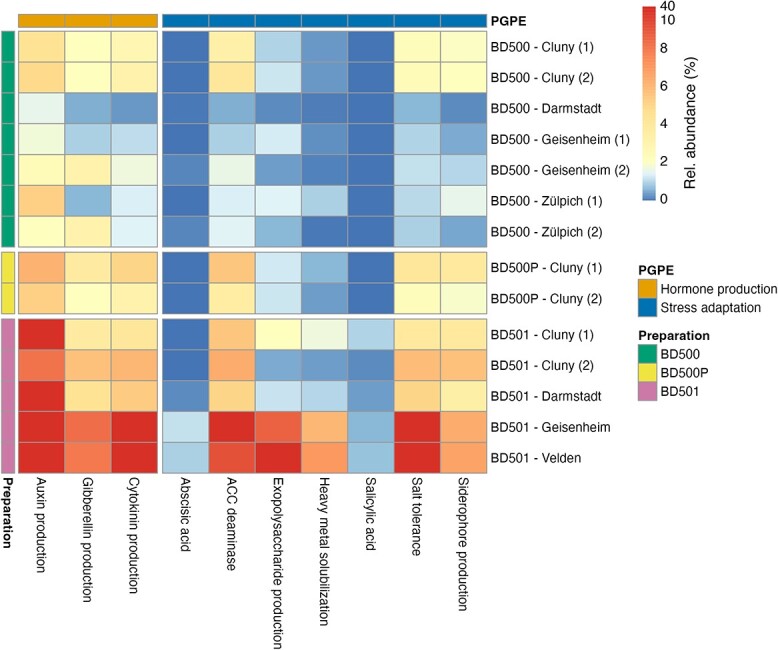
Heatmap of relative abundance of ASVs that perform putative plant growth promoting functions according to the BeCrop databases for all sequenced biodynamic preparations. Biodynamic preparations are separated by horn-manure preparations (BD500 and BD500P) and horn-silica preparations (BD501). The cities where biodynamic preparations were produced are displayed as row labels. Multiple preparations were sampled in some cities, which is denoted with numbers after city labels. Preparations from Velden and Zülpich were not applied at the experimental sites but were included in the analysis to account for the variability of PGPE of biodynamic preparations.

We sequenced several biodynamic preparations used in the experimental trials (Cluny, Geisenheim, Darmstadt), but also additional preparations from other biodynamically managed farms in Germany (Zülpich, Velden) to account for location-specific variation in microbiomes. We sequenced several preparations of the same kind (BD500, BD500P, BD501) for which we estimated relative abundances of prokaryotes and fungi that induce potential PGPEs based on the databases of Biome Makers (Fig. 4). The potential PGPEs were differentially abundant between the two major preparation types with and without manure, similar to their community differentiation. The highest relative abundance of potential PGPE promoting organisms was found in preparations based on horn silica (BD501), whereas preparations that used manure (BD500 and BD500P) exhibited generally lower relative abundances. Especially the abundance of potentially hormone producing microorganisms was considerably high: up to 47% of the microbiome in the preparation from Velden could potentially synthesize auxin. This sample exhibited generally high relative abundances of organisms that potentially perform PGPEs. Overall, potentially hormone producing prokaryotes and fungi were enriched in horn silica preparations and to a lesser extent also in the manure preparations. Potentially stress adaptation promoting microorganisms were on average rarer than hormone producing organisms. Their most prominent effects were increased salt tolerance, ACC deaminase, and EPS production. ABA and salicylic acid producing microorganisms were nearly absent from the preparations and constituted only minor community proportions, regardless of location and preparation type.

### Time-dependent plant growth promoting effects of biodynamic preparations

**Figure 5:**
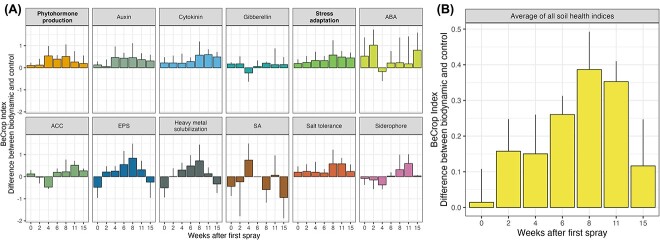
Time series analysis of putative plant growth promoting functions performed by soil microbial communities. Functional abundance is represented in the barplots by the difference of the BeCrop index from Biome Makers between biodynamically and organically treated soils. Positive values denote higher index values in the biodynamic treatment and negative values vice versa. The BeCrop index scales with abundance of microbial organisms that promote individual plant growth promoting functions and varies from 1 to 6. Weeks after the first spray treatment in the time series are shown on *x*-axes. Microbial functions that promote plant growth are grouped into hormone production (phytohormones) and stress adaptation. Functional groups are displayed in bold text. Time series of all inferred plant growth promoting functions are denoted in (A) and the mean index differences of all functions are displayed in (B). Error bars denote standard error.

The time series analysis conducted at two different locations (in Germany and France, see Table 1) yielded similar potential PGPEs that were enriched in treatments as found in the other experiments. We analyzed the difference of potential PGPEs between control and treatment for the individual locations with positive values indicating an enrichment and negative values indicating a depletion of potential PGPE conducting microorganisms (Fig. 5). The three fields in France were sprayed only once with horn manure and horn silica, whereas the field in Germany was sprayed four times with horn manure in the beginning of the experiment and twice with horn silica thereafter. Several indices showed a strong increase in biodynamic treatments compared with the controls in the field trials, such as auxin, cytokinin, and EPS production (Fig. 5), whereas others did not exhibit significant differences between control and treatment in the field trials (gibberellin and SA production) (Fig. 5A). Altogether, plant growth promoting functions expressed a recurrent mean pattern with increasing values at the start of the treatment with the biodynamic preparations until 8 weeks after the spray treatment. Thereafter, the mean values of potential PGPEs decreased again, indicating that control and treatment indices converged (Fig. 5B). This pattern was similar to the pattern of colonization success reported earlier (Fig. 2B).

## Discussion

Our results indicate that the application of biodynamic preparations on agriculturally used soils has implications on the resident soil microbiota. Our experimental design to assess the impact of management practice on microbial soil communities covered a broad range of regions within France and central Germany, crops, timepoints, and farms, each offering different soil properties. Our data consistently support our initial hypotheses across diverse setups, underlining their validity. We found that (i) the application of biodynamic preparations has an effect on the microbial community composition and (ii) communities are mainly affected by an increase of ASVs that were also enriched in the biodynamic preparations. Furthermore, (iii) biodynamic preparations were composed to a high extent of putative plant growth promoting organisms and its application increased the abundance of putative PGPM in soil communities. However, (iv) our time series analyses show that putative PGPM are enriched with a maximum after 8 weeks and decreasing values thereafter in biodynamically treated soils compared with organically treated soils.

### Microbial variability in agriculturally used soils

The prokaryotic and fungal communities sequenced showed a highly similar taxonomic composition on genus level among all experimental sites. However, ASVs of the same taxonomic groups strongly differed between samples, indicating species or subspecies diversification. Taxonomic composition of fungi varied much stronger compared with prokaryotes, which is in agreement with previous studies that found neutral (i.e. stochastic) processes to be more important for fungal community assembly as compared with prokaryotic communities [39, 40]. The variability of ASVs followed mainly farm location and sampling timepoints, whereas agricultural management and crops had a much lower impact on the resident soil communities. Marginal differences between microbial community compositions of organically and biodynamically treated soils relative to other factors were also found by other studies [25, 41]. Microbial soil communities are highly diverse, with thousands of different organisms found within a single sample [42] and whose composition and diversity are strongly shaped by climate [43, 44] or pH [45]. Nonetheless, cropping practice has a measurable impact on microbial community composition, driven e.g. by tillage [46] or type of fertilizer [41], but its effect on the microbial biogeography in soils is minor compared with the before-mentioned drivers [46]. Therefore, we traced mainly those ASVs enriched in biodynamic preparations to minimize variation induced by other factors. We found an overall significant increase of ASVs in soil communities enriched in biodynamic preparations, revealing a direct effect of management practice on the studied soil communities. Increasing the spray frequency of biodynamic preparations further enhanced the abundance of these ASVs, indicating that biodynamic preparations can act as vessels for biological soil amendments [47]. Our time series analyses showed that biodynamic preparation associated ASVs were most abundant 8 weeks after first inoculation, declining afterwards. Survival time of so called biofertilizers typically ranges in the order of weeks and is highly dependent on soil properties [48] and biotic interactions with the resident soil community [49].

### Plant growth promoting microorganisms in biodynamic preparations

As stated before, it is assumed that biodynamic preparations influence microbial soil communities via two independent mechanisms: (i) microbial activation via signaling molecules that accumulate in the fermented products [16] and (ii) successful colonization of plant growth promoting organisms that reside in communities associated to the biodynamic preparations. Our results indicate high abundances of putative PGP fungi and prokaryotes in the biodynamic preparations that produce phytohormones such as auxin, but also perform stress reducing actions, such as solubilization of heavy metals or production of EPS. We detected higher abundances of putative PGP organisms in the preparations containing silica powder (preparation BD501) instead of manure (preparation BD500) represented by high abundances of Gammaproteobacteria, Actinobacteria, and Eurotiomycetes. Generally, horn silica preparations harbored different communities compared with horn manure preparations that were dominated by Clostridia and Alphaproteobacteria on 16S rRNA gene level and Morteriellomycetes on ITS level. Our results match the results of other studies [22, 23], which also found high abundances of potentially plant growth promoting genera in manure- and plant-based biodynamic preparations, such as *Mortierella*, *Penicillium,* and *Aspergillus*. The fermentation and ripening of biodynamic preparations in soils lead to the accumulation of biolabile components and undecomposed lignin compounds [16]. Similar growth promoting effects have been found for composted tea preparations [50] and water extractable organic matter from different compost preparations [51]. Hence, we hypothesize that the effect of biodynamic preparations on soils might be similar to biological amendments, such as compost, straw, or biochar, that have a direct impact on microbial soil communities. They increase microbial enzyme activity, biomass, and soil respiration [52]. Based on our results, we assume that biodynamically managed soils differ from organically managed soils because of higher abundances of putative plant growth promoting microorganisms that are introduced via biodynamic preparations, together with biolabile compounds that can have stimulating effects on resident communities.

### Effect of biodynamic preparations on soil microbial communities

We found evidence that biodynamic preparations increase the abundance of organisms that potentially promote biostimulation of plants via production of phytohormones (auxin, cytokinin, and gibberellin). Furthermore, organisms that protect crops from biotic and abiotic stressors via mechanisms such as siderophore production or increasing salt tolerance were also increased in biodynamically treated soils. Biodynamic preparations seem to enhance the abundance of microbial organisms that act on such a broad functional spectrum. Organisms that are known to have plant growth promoting properties often perform multiple beneficial functions, such as strains of the species *Bacillus subtilis* whose plant growth promoting activity has been intensively studied [53]. This bacterial group enhances plant growth by improving nutrient availability, altering plant growth hormone homeostasis and reducing drought and salt stress [53]. Therefore, a simultaneous increase of multiple PGP effects is likely, especially because we inferred putative microbial functions based on taxonomic identities. We conclude that the general trend of increased PGP functions in biodynamically managed soils reflects high abundances of putative PGP organisms.

The time series data showed increased PGP functions in soil communities that matched the before-mentioned colonization patterns of microbes. We further identified low colonization success of microbes associated with biodynamic preparations in soils from Frankenhausen that were sampled 16 weeks after the first spray treatment. Assuming the strongest effect of biodynamic preparations 8 weeks after first treatment, our sampling strategy in Frankenhausen might have missed significant changes in microbial community composition. Microbial soil inoculants face strong selective pressure after colonization, especially in the rhizosphere [54]. Inoculation of microbes directly on the field can affect the resident soil communities [49] and is therefore used in commercial products to enhance crop yield [55] or protect plants from disease outbreaks [56, 57]. Such biofertilizer typically affect microbial communities in timeframes of weeks after which the inoculated strains decline in abundance [58, 59].

Microbially mediated plant growth promotion through application of biodynamic preparations has been assumed in other studies that detected putative PGP organisms in biodynamic preparations [22, 23]. However, this study provides first evidence that such mechanisms will be enhanced through biodynamic crop management compared with organic crop management because of successful colonization of plant growth promoting organisms via biodynamic preparations. The fact that our results were derived from field studies stresses their relevance for decisions in agriculture, but further experiments are necessary to identify which PGP effects are enriched on a genomic level and how they affect plant growth.

Geisenheim stood out in our field trials as it was the only setup that did not express increased PGP effects in soil microbial communities that were biodynamically managed. Instead, the trend was vice versa with generally lower abundances of putative PGP organisms. The vineyard in Geisenheim has every second year high leguminous cover crops that promote higher nitrogen availability for plants in organically and biodynamically than in conventionally managed soils [60] and therefore stands out from the other experimental setups. A generally high nutrient availability might reduce the enrichment of PGP organisms via selective colonization at the plant–soil interface [61], since the plant will less likely select for biofertilizing symbionts [62, 63]. This is in accordance with the previously mentioned study that found increased crop yield after application of biodynamic preparations under unfavorable growth conditions, whereas under high nutrient supply crop yield was not affected or even reduced [15]. Hence, we assume that biodynamic preparations are compensatory with strongest positive effects on plant growth under unfavorable conditions, consistent with selective colonization at the plant–soil interface.

### Biodynamic preparations as biological amendments of soils

Studies that analyzed microbial soil properties with respect to agricultural management found the highest soil microbial biomass and the lowest ratio of microbial respiration to biomass in biodynamically managed soils [5, 64]. Furthermore, biodynamic management promotes densely connected co-occurrence networks in soil microbial communities that represent collaborative communities [25]. How biodynamic preparations work and under which circumstances remains elusive, alike other microbial inoculants [57]. Previous studies found varying effects of inoculated PGP microorganisms, depending e.g. on soil nutrient availability [65] or organic matter content [66]. Similarly, the application of biodynamic preparations led to significant increases in soil activity and crop yield [6] but in some cases yielded no significant effects [13, 14]. Plant growth beneficial effects of biodynamic preparations have been detected before and were most pronounced under unfavorable plant growth conditions [11, 15]. We found evidence for plant beneficial changes in microbial community composition in various soil types (haplic Luvisol, clay, loam, sandy loam) in Germany and France and for various crops (grapevine, oats, spelt, wheat, chickpeas, rye, barley, garlic, flax, sunflower). Since the plant beneficial effects are microbially mediated, we assume that further insight into bacteria-plant interactions is required to improve our understanding under which conditions biological amendments have measurable beneficial effects. Also, while the sum of these effects might promote soil health, their implications on crop yield and quality remain uncertain [67]. Therefore, further studies should focus on the phyllosphere and rhizosphere where microbes from the spray treatment can establish and interact with plants and promote their growth [68, 69]. Metagenomic and metatranscriptomic analyses are necessary to verify not only the genomic potential of inoculated strains, but also whether their plant growth promoting functions are expressed and under which conditions.

## Supplementary Material

Supplementary_Material_ISME_Comm-Revised_ycae021

Supplementary_Data-REVISION_ycae021

## Data Availability

The 16S rRNA gene and ITS reads have been deposited at ENA under accession nr. PRJEB65929. Associated sample meta-data and BeCrop indices are provided as supplementary data. All R scripts to reproduce analyses are uploaded to GitHub (https://github.com/dermilke/Biodyn).
