# Supplementary material for: Enrichment of putative plant growth promoting microorganisms in biodynamic compared with organic agriculture soils
Source: ISME Commun. 2024 Feb 5;4(1):ycae021. doi: 10.1093/ismeco/ycae021 (PMC11656761; doi:10.1093/ismeco/ycae021)
Supplement: Supplementary_Material_ISME_Comm-Revised_ycae021 [file supplementary_material_isme_comm-revised_ycae021.docx]

**Supplementary Information**

**Description of experimental sites**

Geisenheim: A long-term field trial on grapevine has been conducted at Geisenheim University (49°59.326'N, 7°56.813'E) since 2006, comparing conventional, organic, and biodynamic farming systems (1,2). The experimental site is a 0.8 ha vineyard (*Vitis vinifera* L. cv. Riesling) at Geisenheim, Germany (49° 59.326'N, 7° 56.813'E). The vines were planted in 1991. Since January 2006 the vineyard has been cultivated according to the rules of integrated (GAP), organic and biodynamic viticulture (both regulations EU 834/07 and 889/08). The difference between organic and biodynamic cultivation is solely the application of the biodynamic preparations. The trial was set up as a complete block trial with four replicates. Each plot comprises four rows with 32 vines. The average annual temperature is 11 °C, the altitude is 137 m above sea level (asl) and the mean precipitation is 527 mm per year. For further information, see Meissner et al. (2).

Frankenhausen: The long-term trial at Frankenhausen is located near Kassel, Germany (51° 24.590’ N, 9° 26.053' E). The trial is managed organically and was established with different crop rotations and fertilization variants in 2017. The soil contains 18.48% clay, 79.95% silt, and 1.57% sand in the Ap horizon. The soil type is a haplic luvisol with a large loess layer. The average annual temperature is 8.5 °C and the average precipitation 650 mm per year. In the field trial, three livestock-free farming systems and one traditional farming system with livestock are mimicked with typical crop rotations and fertilization strategies and compared with each other (3). Since 2021, biodynamic spray preparations have been applied as a factor with and without treatment in four treatments of the long-term trial in four field replications each, resulting in 16 plots. The trial is therefore designed as a two-factor split-plot system, with the farming systems as main plots (soil fertility farm with manure, crop 2021: oats, soil fertility farm with green waste compost, crop 2021: oats, vegan farm without fertilization, 2021: spelt, catch crop farm with biogas residues, 2021: wheat) and the application with and without the biodynamic spray preparations as subplots.

Darmstadt: The experimental area at Darmstadt (8° 41.683' E 49° 53.733' N) has been farmed biodynamically for 15 years. The soil is a haplic luvisol with a sandy texture (88.8 % sand, 6.4 % silt, and 4.8 % clay). The experimental area is 170 m asl and has a mean annual precipitation of 692.5 mm and a mean annual temperature of 10.6 °C.

The field trial was set up in 2019 as a randomized split plot with four replications. The main plots were different pre-crops in 2019 and 2020 (lucerne, tall fescue, lucerne-tall fescue mixture, cereals). The following crop was spring wheat in 2021. Subplots varied in application frequency of the biodynamic preparations: i) 1 time horn manure and 1 time horn silica, and ii) 3 times horn manure, 3 times horn silica and additionally application of the biodynamic compost preparations in the rotting of cattle deep stable manure (Supplementary table S1). Deep stable manure was applied in two heaps, the minimum variant was treated without compost preparations, and the heap for the variant with multiple preparation applications was treated with the compost preparations. Deep stable manure was applied with 100 kg N ha^-1^ a^-1^.

France: This trial involved 21 agricultural sites spread over France cultivated organically. For the experiment, the sites were split in two halves: one half applying biodynamic preparations and the other one not. Grapevine are grown at 13 of the 21 sites, chickpeas at two sites, and rye, barley, garlic, wheat, flax, and sunflower at one site each. The soil texture is clay at 13 of the 21 sites, loam at 4 sites, and sandy loam at 4 sites. More details on the individual sites, such as location, soil properties, preparation applications, and since when biodynamic management was applied are described in supplementary table S2. The different sites were treated as replicates and the treatments with and without preparations were compared at the respective sites.

Time series: Three sites are located in the village of Mérignat near Bourg-en-Bresse in France. Mérignat location A (46° 04.184' N, 5° 25.888' E) has clay soil and lies at 380 m asl. Mérignat location B (46° 04.275' N, 5° 26.518' E) has clay soil and lies at 390 m asl. Mérignat location C (46° 04.043' N, 5° 26.640' E) has silty-clay soil. The location is at 446 m asl. In Mérignat the average annual temperature is 13.2 °C and the mean precipitation is 1043 mm per year. The fourth location is at the long-term field trial in Frankenhausen near Kassel in Germany (see above). The four experimental sites from the time series were treated with biodynamic preparations for the first time in 2021. We evaluated the different sites as replicates for the time series analysis, as each site was treated with and without preparations and soil was sampled before spray-treatment and 2, 4, 6, 8, 11, and 15 weeks after first treatment.

1. Döring J, Frisch M, Tittmann S, Stoll M, Kauer R. Growth, Yield and Fruit Quality of Grapevines under Organic and Biodynamic Management. Kurtural SK, editor. PLoS ONE. 2015 Oct 8;10(10):e0138445.

2. Meissner G, Athmann ME, Fritz J, Kauer R, Stoll M, Schultz HR. Conversion to organic and biodynamic viticultural practices: impact on soil, grapevine development and grape quality. OENO One [Internet]. 2019 Oct 18 [cited 2023 Jul 5];53(4). Available from: https://oeno-one.eu/article/view/2470

3. Möller M, Dreßen S, Athmann M, Bruns C. Langzeitversuch zu viehlosen Betriebssystemen im ökologischen Landbau. In Frick (CH): 16th Scientific Conference on Organic Agriculture; 2023. Available from: https://orgprints.org/id/eprint/50560/

Table S1: Detailed description of treatments for each experimental setup, including timepoints of spraying of biodynamic preparations and their frequency. Treatment 1 was used as control and treatment 2 as treated sample groups. Additional information on experimental sites in France can be found in supplementary table S2. Number of replicates were: Frankenhausen N = 16, France N = 21, Darmstadt N = 16, Geisenheim N = 4.

| **Location** | **Treatment 1 .** | | **Treatment 2 .** | |  |
| --- | --- | --- | --- | --- | --- |
|  | **Variant 1** | **Spraying variant 1** | **Variant 2** | **Spraying variant 2** | |
| **Darmstadt** | Biodynamic | 1 x hornmanure 9.3.2021 | Organic with BD | 3 x hornmanure (2.3.;9.3.;27.3.2021) | |
|  | extensiv | 1 x hornsilica 8.6.2021 | prep. (BD++) | 3 x hornsilica (8.6.;21.6.;15.7.2021) | |
|  |  |  |  |  | |
| **Geisenheim** | Organic | no biodyn prep. | Organic with BD | 3 x hornmanure (25.3., 3.5., 8.11.2021) | |
|  | (BD-) |  | prep. (BD++) | 3 x hornsilica (17.6., 25.8., 5.10.2021) | |
|  |  |  |  |  | |
| **Frankenhausen** | Organic | no biodyn prep. | Organic with BD | 4 x hornmanure (28.+ 29.+ 30.4.+1.5.2021) | |
|  | (BD-) |  | prep. (BD++) | 2 x hornsilica (29.+31.5.2021) | |
|  |  |  |  |  | |
| **France** | Organic | no biodyn prep. | Organic with ext. | 1-2 hornmanure (15.4. - 30.4.2021) | |
|  | (BD-) |  | BD prep. (BD+) | 1-3 hornsilica (20.5. - 28.9.2021) | |
|  |  |  |  |  | |
| **Time series** |  |  |  |  | |
| Mérignat location A | Organic | no biodyn prep. | Organic with ext. | 1 x hornmanure (24.4.2021) | |
|  | (BD-) |  | BD prep. (BD+) | 3 x hornsilica (29.5.+18.7.+6.9.2021) | |
| Mérignat location B | Organic | no biodyn prep. | Organic with ext. | 1 x hornmanure (24.4.2021) | |
|  | (BD-) |  | BD prep. (BD+) | 3 x hornsilica (29.5.+18.7.+6.9.2021) | |
| Mérignat location C | Organic | no biodyn prep. | Organic with ext. | 1 x hornmanure (24.4.2021) | |
|  | (BD-) |  | BD prep. (BD+) | 3 x hornsilica (29.5.+18.7.+6.9.2021) | |
| Frankenhausen D | Organic | no biodyn prep. | Organic with BD | 4 x hornmanure (28.+ 29.+ 30.4.+1.5.2021) | |
|  | (BD-) |  | prep. (BD++) | 2 x hornsilica (29.+31.5.2021) | |

Prep: preparation

Table S2: Additional information about each sample site in France, including location, crops, soil type and applied biodynamic preparations.

| **Location** | **Coordinates** | **Fruit in 2021** | **Soil type** | **Spraying 2021** | **prep. since** |
| --- | --- | --- | --- | --- | --- |
| 1 | 46°17.019'N 4°46.264'E | Vine | Clay | 1 x 500P + 2 x 501 | 2019 |
| 2 | 46°19.161'N 4°43.598'E | Vine | Clay | 2 x 500P + 1 x 501 | 2018 |
| 3 | 46°19.161'N 4°43.598'E | Vine | Clay | 2 x 500P + 1 x 501 | 2018 |
| 4 | 46°11.486'N 4°40.501'E | Vine | Sandy loam | 2 x 500P + 3 x 501 | 2016 |
| 5 | 46°15.576'N 4°45.209'E | Vine | Clay | 2 x 500P + 2 x 501 | 2007 |
| 6 | 46°15.576'N 4°45.209'E | Vine | Clay | 2 x 500P + 2 x 501 | 2007 |
| 7 | 46°31.010'N 4°44.609'E | Vine | Clay | 2 x 500P + 3 x 501 | 2016 |
| 8 | 46°31.010'N 4°44.609'E | Vine | Clay | 2 x 500P + 3 x 501 | 2016 |
| 9 | 46°16.032'N 4°45.960'E | Vine | Clay | 2 x 500P + 1 x 501 | 2001 |
| 10 | 46°16.032'N 4°45.960'E | Vine | Clay | 2 x 500P + 1 x 501 | 2001 |
| 11 | 48°14.108'N 7°21.520'E | Vine | Sandy loam | 2 x 500 P + 4 x 501 | 2018 |
| 12 | 47°15.718'N 1°16.828'E | Rye | Sandy loam | 1 x 500P + 1 x 501 | 2020* |
| 13 | 43°42.787'N 0°57.211'E | Chickpeas | Sandy loam | 1 x 500P + 1 x 501 | 2020* |
| 14 | 43°42.556'N 1°01.205'E | Barley | Loam | 1 x 500P + 1 x 501 | 2020* |
| 15 | 43°45.033'N 0°39.882'E | Chickpeas | Loam | Summer 2021 1 x 501 | 2020* |
| 16 | 43°54.533'N 0°47.283'E | Garlic | Clay | Summer 2021 1 x 501 | 2020* |
| 17 | 43°57.041'N 0°23.660'E | Wheat | Clay | 1 x 500P + 1 x 501 | 2020* |
| 18 | 43°57.041'N 0°23.660'E | Brown flax | Loam | 1 x 500P + 1 x 501 | 2020* |
| 19 | 46°27.653'N 4°34.191'E | Vine | Clay | 1 x 500P + 1 x 501 | 2020* |
| 20 | 46°27.653'N 4°34.191'E | Vine | Clay | 1 x 500P + 1 x 501 | 2020* |
| 21 | 43°36.063'N 0°54.520'E | Sunflower | Loam | 1 x 500P + 1 x 501 | 2020* |

Prep: preparation; 500P: hornmanure with compostpreparation, 501: hornsilica;
2020*: November 2020 1 x 500P

Table S3: Effect of biodynamic treatment on abundance of plant growth promoting functions quantified by BeCrop index values. The relative difference of BeCrop indices between biodynamic and control treatment is colour coded according to the legend. Total number of functions that show an increase and decrease in the treatment relative to the control are shown in the bottom rows. Symbols represent statistical significance: T = p-value < 0.1, * = p-value < 0.05, ** = p-value < 0.01.


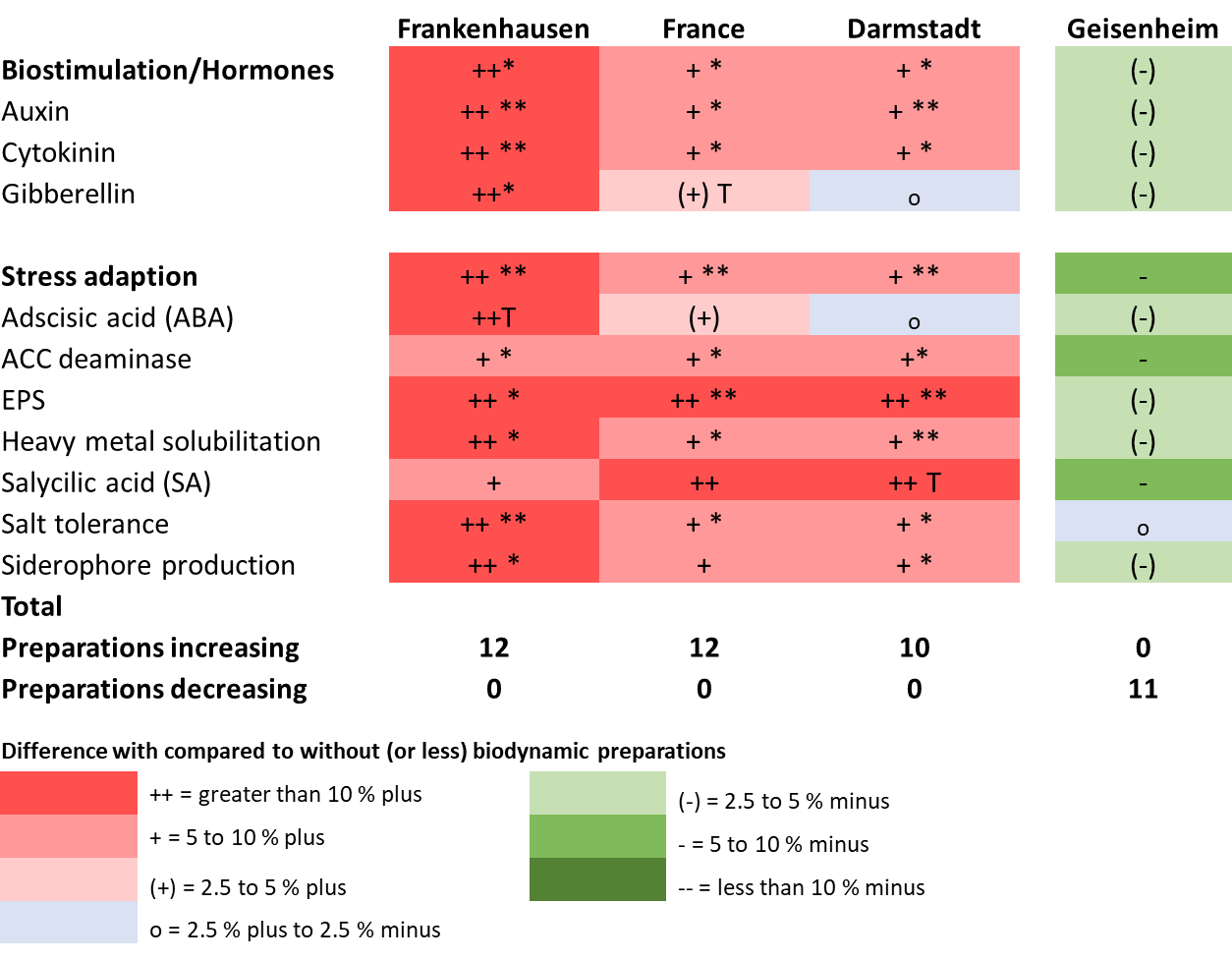


Table S4: BeCrop indexes list and definition grouped by their respective category.

| **BeCrop® index category** | **BeCrop® index** | **Description** |
| --- | --- | --- |
| Hormones Impact | Auxin production (IAA) | Responsible for cell division and elongation |
|  | Cytokinin production (CK) | Responsible for cell proliferation and differentiation |
|  | Gibberellin production (GA) | Responsible for elongation, germination and flowering |
| Stress Impact | Abscisic acid (ABA) | Growth regulation, Plant resistance |
|  | ACC deaminase (ACC-d) | Pathogen protection. Drought protection |
|  | Exopolysaccharide production | Nutrient trap, Salinity protection |
|  | Heavy metal solubilization | Bioremediation, Detoxification |
|  | Salicylic acid (SA) | Alleviate water stress, Salinity protection |
|  | Salt tolerance | Alleviate water stress, Root Growth |
|  | Siderophore production | Iron nutrition, Biofertilization |

Figure S1: Validation of BeCrop index values using database built on literature review. Biome Maker index values for hormone production (**A**) and stress-adaptation (**B**) are displayed against predicted function abundance. Predicted function abundance is based on a linear model that uses abundance of putative plant growth promoting prokaryotes and fungi. The model performance and significance is shown in the respective figures.

Figure S2: Taxonomic community composition. Barplots showing average community composition of different locations, management types and timepoints (T0 and T1, see Table 1 for details). Taxonomic assignments are displayed by genus and colour coded according to the legend. Taxonomic genera below 0.9 % relative abundance are grouped into others. Subfigure **a)** shows prokaryotic community composition and **b)** shows fungi community composition.

Figure S3: Prokaryotic and fungal community variation. NMDS ordination plots for soil prokaryotic **(a)** and fungi **(b)** communities. Colours display sampling locations and shapes represent sample type. Samples are separated by sampling timepoints T0 and T1. NMDS stress values are shown in the plots.

Figure S4: Nonmetric multidimensional scaling of microbial communities in biodynamic preparations. Abundance data was Bray-Curtis transformed prior to ordination. Prokaryotic (**A**) and fungal (**B**) communities are coloured by the biodynamic preparation they were originating from, and the shape denotes the countries where biodynamic preparations were prepared.

Figure S5: Fungi communities enriched in biodynamic preparations and their abundance in soils. **A** Composition of fungi enriched in biodynamic preparations from various locations and preparation types. ASVs are defined to be enriched in biodynamic preparations if they have relative abundance higher than 0.5 %. Taxonomic assignment is displayed at genus level and colour coded according to the legend. **B** Abundance difference of fungi ASVs enriched in biodynamic preparation between treatment and control. Positive values indicate higher abundance of ASVs in treated soils.
